# Locked Cerclage Wiring for Distal Clavicle Fractures: Surgical Technique and Clinical Outcomes in 11 Patients

**DOI:** 10.7759/cureus.91684

**Published:** 2025-09-05

**Authors:** Kazuhiro Ikeda, Yuuki Mitani, Takamasa Kudo, Hiromitsu Tsuge, Shotaro Teruya, Noya Kikuchi, Sho Kohyama, Shinzo Onishi, Takaji Yanai

**Affiliations:** 1 Department of Orthopedic Surgery, Institute of Medicine, University of Tsukuba, Tsukuba, JPN; 2 Department of Orthopedic Surgery, Kikkoman General Hospital, Noda, JPN

**Keywords:** cerclage wiring, distal clavicular fracture, surgical outcome, surgical technique, tension band wiring

## Abstract

Background*:* Cerclage wiring (CW) is a surgical option in distal clavicular fractures with small bone fragments; however, complications associated with pin backout are occasionally observed. Therefore, we introduced a locked CW system integrating the pin and sleeve and evaluated its clinical outcomes.

Methods*:* This case series included 11 patients who underwent locked CW fixation (age, 57.5 ± 20.0 years; eight men, three women). Postoperatively, shoulder range of motion exercises were commenced at two weeks based on pain tolerance. The primary outcomes were the range of forward elevation, the Constant and American Shoulder and Elbow Surgeons (ASES) scores at six months, bone union rates, pin backout distances, and postoperative complications.

Results*:* The range of forward elevation was 176.7 ± 22.2° at six weeks and 180 ± 0° at six months postoperatively. The Constant and ASES scores were 100 (95-100) and 95 (95-100), respectively. Bone union was achieved in all cases. Compared with immediately after surgery, the pin backout distance was 0.9 mm (-0.2 to 1.2 mm). One patient developed a surgical site infection. Two patients had subcutaneous pin prominence that required implant removal.

Discussion*:* Locked CW showed excellent early recovery of shoulder motion after surgery. This outcome may be attributed to stable fixation even in small distal fragments, minimal pin backout, and the ability to initiate range-of-motion exercises at an early stage.

Conclusion*:* Locked CW appears to be a useful and reliable option for distal clavicular fractures. Further comparative and multicenter studies are needed to validate these results.

## Introduction

Distal clavicular fractures account for 10-20% of all clavicular fractures [1]. Among these, fractures involving disruption of the coracoclavicular ligament (Craig classification types II, IV, and V) often require surgical intervention due to the superior and posterior displacement of the proximal fragment [1-3]. Surgical methods include locking plate fixation, which bridges the bone fragments of the clavicle [4,5]; hook plate fixation and coracoclavicular ligament reconstruction, which stabilize the proximal fragment and scapula [6-8]; and cerclage wiring (CW), which directly fixes the bone fragments [7,9,10]. Maintaining anatomical reduction with any of these surgical methods leads to favorable bone union rates [9-12].

However, achieving stable fixation becomes increasingly challenging as the distal fragment size decreases. Locking plates require sufficiently large bone fragments for the insertion of multiple screws, making them unsuitable for fractures with small distal fragments. Hook plate fixation is adaptable irrespective of fragment size; however, its non-physiological immobilization of the acromioclavicular joint increases the risk of acromial bone erosion, acromioclavicular joint arthritis, and periprosthetic fractures. Consequently, its outcomes are often inferior to those of other surgical methods [7,11-14]. Coracoclavicular ligament reconstruction has demonstrated favorable outcomes in some reports; nonetheless, it is technically demanding and associated with a complication rate of 13%-27%, including loss of reduction and clavicular or coracoid fractures due to bone tunnel creation [11,12,14]. CW enables fragment fixation regardless of fragment size and facilitates an early range of motion (ROM) recovery [7]. Nevertheless, conventional CW is associated with a high complication rate due to pin backout, which occurs in up to 20% of cases. Pin backout can lead to reduction loss, nonunion, and pin protrusion-related infections, limiting the reliability of this technique [7,9,10]. The treatment of distal clavicular fractures with small distal fragments remains particularly challenging, as existing fixation techniques have significant limitations in these cases.

To address these challenges, we introduced a locked CW technique using a pin-sleeve system (AI wiring system; Aimedic MMT, Tokyo, Japan). This system integrates the pin and wire through implant design or wiring techniques, aiming to reduce the risk of pin backout and improve fixation stability [15,16]. This study aimed to evaluate the clinical outcomes of the locked CW procedure in patients with distal clavicular fractures, particularly in cases involving small distal fragments.

## Materials and methods

Study design and participants

This study was a retrospective case series based on medical records (evidence level IV). The Ethics Committee of Kikkoman General Hospital approved the study protocol (approval number: KC-H41; date of approval: March 24, 2025). The study details were publicly posted in the outpatient waiting area and on the hospital website for more than five months, and informed consent was obtained via an opt-out process.

This study included patients with distal clavicular fractures who were treated with the locked CW procedure from April 2020 to October 2023. All surgeries were performed by a single surgeon (K.I.) at a single secondary medical center. Surgical indications included Craig classification types II and V, as well as type I in patients who expressed a strong desire for early recovery of activities of daily living [2]. The locked CW procedure was performed in all cases meeting the surgical indications mentioned above. Patients with multiple injuries, including fractures, were excluded.

During the study period, 17 patients with distal clavicular fractures were treated. Among them, conservative treatment was performed in three patients with Craig classification type I. Out of 14 surgically treated patients, three patients with multiple injuries (two with vertebral fractures and one with multiple rib fractures) were excluded. Ultimately, 11 patients with distal clavicular fractures were included in this study.

Treatment protocol

All patients underwent clavicular radiography and computed tomography (CT) on their initial visit, and surgery was promptly performed in eligible cases.

**Figure 1:**
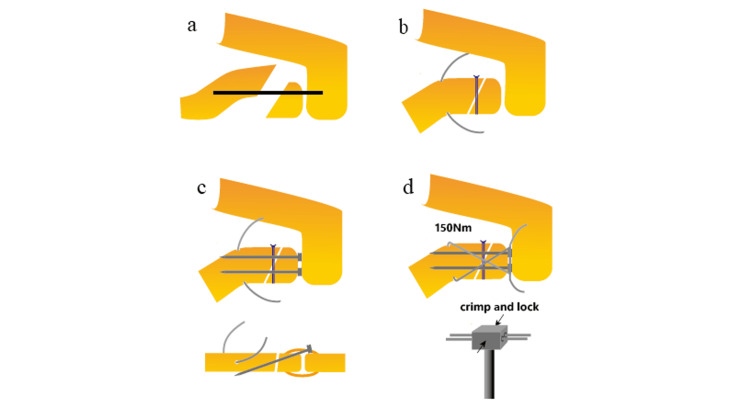
Surgical technique for the locked cerclage wiring procedure. (a) A 5-cm skin incision is made to expose the area from the distal clavicle to the acromioclavicular joint. (b) A 2.0-mm bone hole is created in the proximal fragment at the inflection point of the distal clavicle, and a soft wire is passed through it. After reduction, No. 2 braided sutures are applied to maintain stability in cases of oblique or coracoclavicular ligament avulsion fractures. (c) Two 2.0-mm sleeve pins are inserted into the proximal fragment from the superior aspect of the acromioclavicular joint margin of the distal fragment and are aligned as parallel as possible to the distal clavicle. (d) A figure-eight-shaped soft wire is inserted into the pin–sleeve and tightened to a tension of 150 Nm. As the wire bends at an acute angle, great care must be taken during tightening to avoid kinking. Finally, the sleeve is crimped to lock the soft wire.

**Figure 2:**
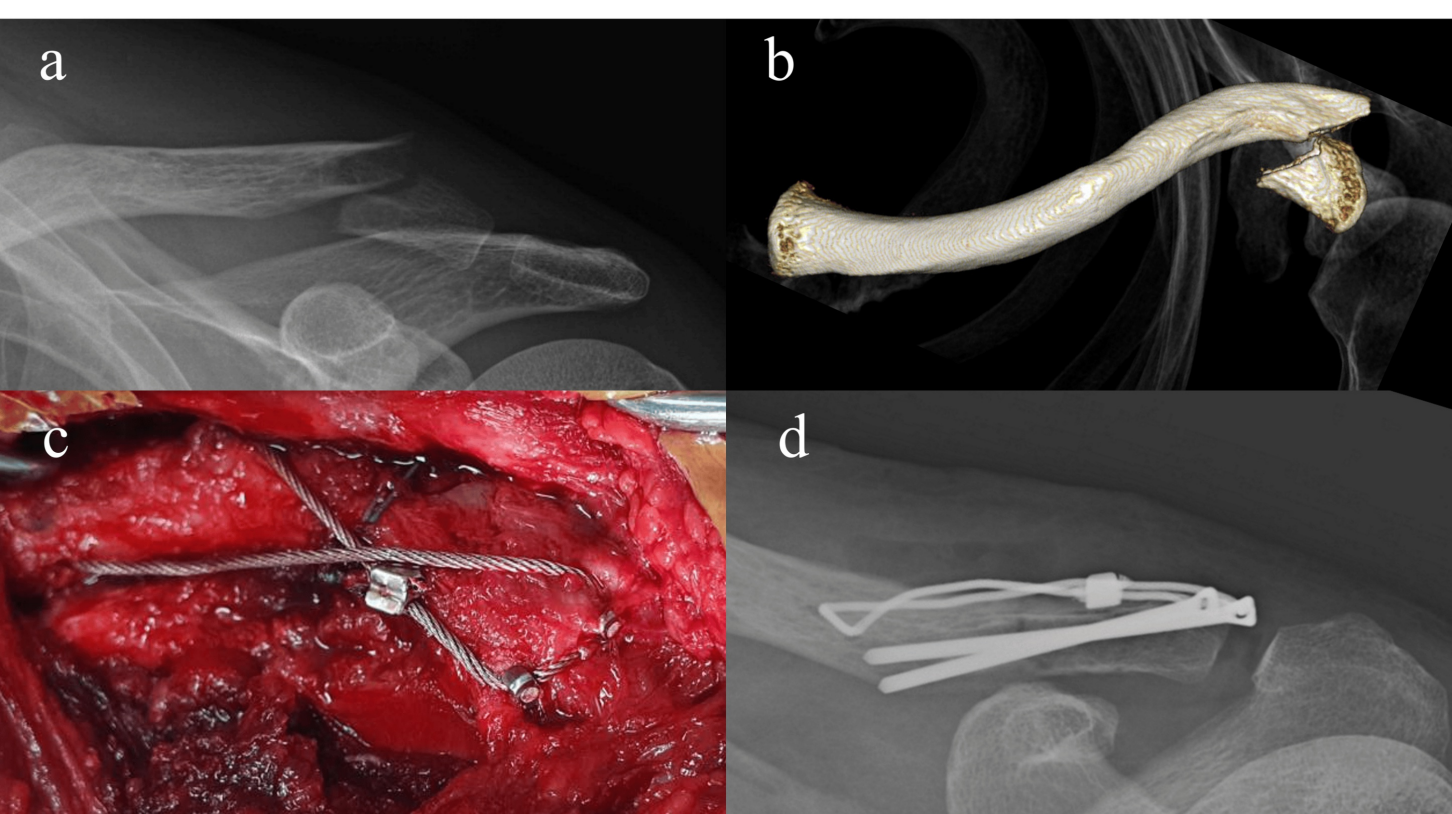
Locked CW for a distal clavicular fracture. (a) Preoperative radiograph showing a distal clavicular fracture. (b) Preoperative three-dimensional computed tomography image showing Craig classification type IIb. (c) Intraoperative image showing locked CW performed, with the pin and sleeve locked. (d) Postoperative radiograph showing satisfactory reduction. CW, cerclage wiring.

The surgical procedure is illustrated in Figure 1, and a representative case is shown in Figure 2. A single orthopedic surgeon (K.I., a shoulder specialist) performed or assisted with the surgeries under general or local anesthesia, depending on the patient's preference.

For the first two weeks after surgery, the arm was immobilized with a sling, and shoulder elevation was restricted to 90°. At two weeks postoperatively, the sling was removed, and shoulder elevation was allowed, as tolerated. From eight weeks postoperatively, patients were permitted to engage in heavy labor. Postoperative follow-up was conducted for a minimum of six months. Follow-up was carried out beyond six months postoperatively until the patients requested discontinuation. For patients with a follow-up period <12 months, functional status was reassessed via telephone.

Outcomes

**Figure 3:**
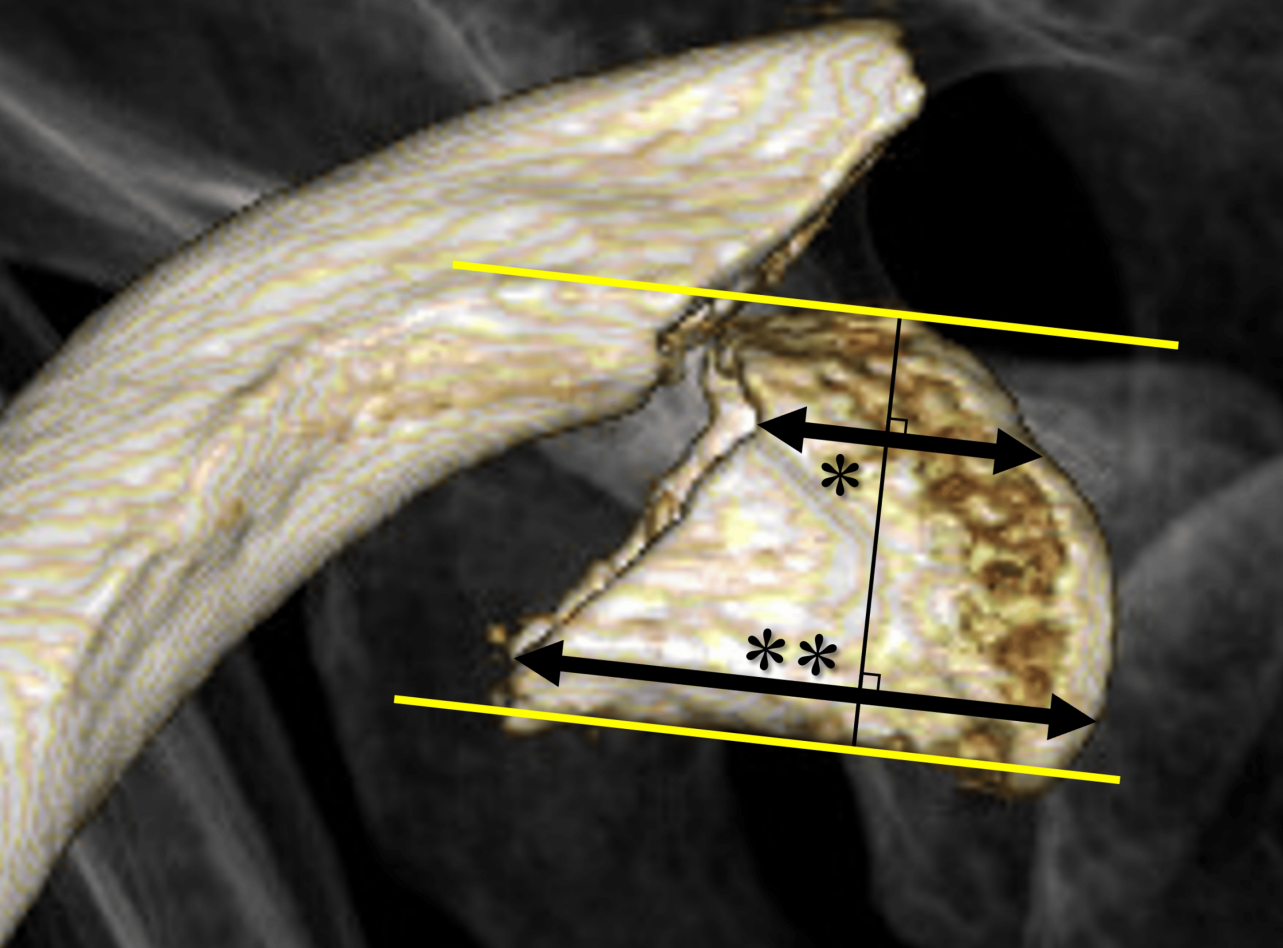
Measurement of the fragment diameter. The fragment size is measured using a superior view of the clavicle obtained from three-dimensional computed tomography. The minimum and max diameters are determined based on parallel lines extending from the fracture site to the acromioclavicular joint along the anterior and posterior cortices of the distal fragment, respectively. The yellow line indicates the outline of the distal clavicle. *, minimum fragment diameter; **, maximum fragment diameter

Patients’ demographic and clinical characteristics, including age, sex, mechanism of injury, fracture type (Craig classification [2]), distal fragment size, surgical waiting time, and postoperative follow-up duration, were analyzed. The distal fragment size was measured using three-dimensional CT images that viewed the clavicle from a superior perspective. The maximum and minimum diameters from the distal end to the fracture site were measured (Figure 3).

The primary measures were the clinical and radiological results of the locked CW procedure. These included operative time, shoulder function at six months postoperatively (ROM and the Constant and American Shoulder and Elbow Surgeons (ASES) scores), bone union, pin backout distance, and postoperative complications. Bone union assessment and measurement of pin backout distance were independently performed by two orthopedic surgeons (Examiner 1: 12 years of experience; Examiner 2: six years of experience). The Constant score, which incorporates both clinician- and patient-reported components, and the ASES score, which is a patient-reported outcome measure, were used to evaluate functional outcomes. Bone union was defined as the presence of cortical continuity or a bridging callus at the fracture site on plain radiographs (anteroposterior and Zanca views), taken six months postoperatively [10]. The pin backout distance was determined by measuring the change in the protrusion length of the pin from the distal end of the clavicle on plain radiographs (anteroposterior view), taken immediately after surgery and six months postoperatively. Each examiner measured the pin backout distance twice, with a one-month interval between measurements. The first measurement taken by Examiner 1 was used for the analysis. Postoperative complications were classified into two categories: major and minor. Major complications included infection, pin cut-out, and nonunion, whereas other complications were categorized as minor.

Statistical analysis

The normality of all continuous variables was assessed using the Shapiro-Wilk test. Normally distributed and non-normally distributed data were presented as mean ± standard deviation and median (interquartile range (IQR)), respectively. The pin protrusion length was compared between preoperative and postoperative measurements using the Wilcoxon signed-rank test. The inter- and intra-rater reliabilities of this parameter were also evaluated for the two examiners. Inter- and intraclass correlation coefficients (ICCs) were interpreted as poor (<0.50), moderate (0.50-0.75), good (0.75-0.90), and excellent (>0.90). All statistical analyses were performed using the Bell Curve for Excel version 4.08 (SSRI Co., Tokyo, Japan), with statistical significance set at a p-value <0.05.

## Results

Demographic and clinical characteristics

The mean patient age was 57.5 ± 20.0 years, and the patients comprised eight men and three women. The mechanisms of injury included falls in 10 patients and traffic trauma in one patient. Based on the Craig classification [2], the fracture types were type I in one patient, type IIb in eight patients, and type V in two patients. The minimum diameter of the fragment was 8.2 (IQR, 6.9-11.4) mm, whereas the maximum diameter was 18.7 ± 6.5 mm. The surgical waiting period was 10.9±4.0 days. The in-office follow-up period was 6.0 (IQR, 6.0-12.8) months, whereas the overall follow-up period was 23.5 (IQR, 12.8-34.1) months.

Clinical outcomes

The details of the surgical information and clinical outcomes are presented in Table 1. The surgery in nine cases was performed under general anesthesia and in two cases was performed under local anesthesia. The operative time was 57.1 ± 18.5 min.

**Table 1:** Demographic and clinical characteristics of the study patients. Pt, patient; y, years; Mini., minimum; Max., maximum; ROM, range of motion; FE, forward elevation; ASES, American Shoulder and Elbow Surgeons score; SSI, surgical site infection *: range of internal rotation was represented by the hand-behind-back level of the vertebrae.

Pt	Age (yrs.)	Sex	Craig type	Fragment diameter		ROM	Clinical score		X-ray outcome			Complication
				Mini.	Max.	FE (°)	ASES	Constant	Bone union	Pin protrusion^**^	Pin back-out^**^ (mm)	
				(mm)	(mm)					(mm)		
1	27	M	IIb	7.2	10.5	180	95	95	+	11.4	0.6	Implant removal
2	80	W	IIb	10.9	23.4	145	100	93	+	11.4	0.5	-
3	53	M	IIb	11.3	24.2	180	100	100	+	4.6	1.3	-
4	60	M	IIb	11.5	20.4	180	100	95	+	11.1	0	Implant removal
5	47	M	V	23.6	31.9	180	90	100	+	5.3	1.8	-
6	28	M	IIb	6.9	10.9	180	100	100	+	7	1.9	-
7	71	W	IIb	12.6	17.8	180	95	100	+	5.3	1	-
8	72	W	IIb	6.8	16.4	180	100	100	+	4.4	0.8	-
9	74	M	V	5	16.8	180	100	100	+	5.1	-0.8	SSI
10	81	M	I	8.2	12.3	180	95	100	+	5.6	1.1	-
11	39	M	IIb	6.9	21.6	180	85	95	+	4.6	-1.1	-

**Figure 4:**
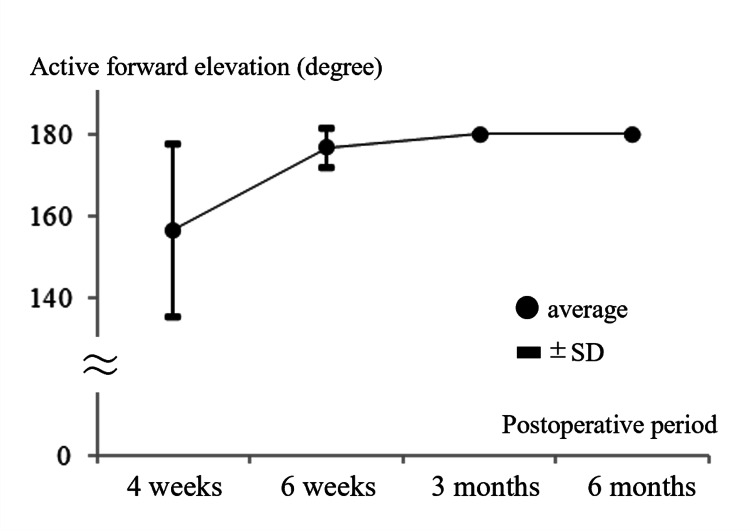
Postoperative changes in the range of forward elevation. The range of forward elevation was nearly fully restored by six weeks postoperatively. SD, standard deviation

The postoperative progression of active forward elevation was as follows: four weeks, 156 ± 22.1°; six weeks, 176.7 ± 5.8°; three months, 180 ± 0°; and six months, 180 ± 0° (Figure 4). The postoperative functional clinical scores were as follows: Constant score, 100 (IQR, 95-100); and ASES score, 95 (IQR, 95-100).

Bone union was achieved in all patients six months postoperatively. The pin protrusion length was 5.0 (IQR, 4.0-9.1) mm immediately after surgery and 5.6 (IQR, 4.8-8.6) mm at six months postoperatively, with a significantly greater protrusion length at six months (p = 0.017). The backout distance was 0.9 (IQR, -0.2-1.2) mm. The agreement rate for bone union assessment was 100%. The ICCs for pin protrusion length were all excellent: ICC (1-1, 1-2) = 0.91 (95% CI, 0.85-0.95); ICC (2-1, 2-2) = 0.90 (95% CI, 0.82-0.94); and ICC (1-1, 2-1) = 0.90 (95% CI, 0.83-0.94) (all p < 0.001). One patient with a history of psoriasis developed a surgical site infection that was managed with wound care and antibiotic therapy. Two patients complained of subcutaneous pin protrusion and pin removal was performed. The protrusion length in these patients was 17 mm and 14 mm.

## Discussion

This study is the first to report the surgical outcomes of the locked CW procedure for distal clavicular fractures, demonstrating favorable postoperative results. Notably, our results indicated rapid recovery of forward elevation range, with near-complete restoration achieved at six weeks postoperatively. Even at this early stage, the ROM exceeded the reported six-month outcomes for other surgical methods: forward elevation of 76-165° with a hook plate [6,17], 165° with tension band wiring [6], 138-160° with a locking plate [4,17], and 167° with a plate and coracoclavicular reconstruction [4]. The excellent ROM may be attributed to the high initial fixation strength between the bone fragments. In the locked CW procedure, the pin directly stabilizes the fragments to maintain the reduction position, whereas the wiring generates a compressive force between the fragments. Additionally, the pin backout force pulled the proximal fragment closer to the distal fragment via the integrated wire, converting this force into compression between the fragments. In fact, the pin backout distance remained at <1 mm over the six-month postoperative period in our cases, which was clinically insignificant. In olecranon fractures, the back-out distance of K-wires was 5.2 mm with conventional CW, 3.7 mm with locked CW without wire-cable integration, and 0.4 mm with locked CW integrating both wire and cable [15,18]. These findings suggest that using a locked CW technique with integrated wire and cable is preferable for enhancing fixation stability and reducing the risk of pin back-out. Excellent initial fixation stability allows patients to begin early postoperative ROM exercises, leading to favorable ROM recovery and functional outcomes.

Another significant advantage of this procedure is its simplicity. The procedure can be completed within an hour, and the limited surgical field allows for day surgery under local anesthesia. The implant cost is generally lower than that of a plate used for distal clavicular fractures (e.g., the Scorpion® NEO, Aimedic MMT, Tokyo, Japan), contributing to reduced healthcare costs. The locked CW procedure imposes minimal burden on patients, physicians, and healthcare systems.

One procedural pitfall is the necessity of embedding the pin as deeply as possible. In our study, two patients with pins protruding by >14 mm requested implant removal because of subcutaneous discomfort and aesthetic concerns. Although embedding the pin deeper brings the sleeve closer to the acromioclavicular joint capsule and makes wire insertion more challenging, insertion depth should not be compromised. Additionally, the acceptable range of pin insertion points is limited because of the small size of the distal fragments. Repeated pin insertions should be avoided to prevent cut-out.

This study has some limitations. As a retrospective study, it was subject to the risks of selection and information biases. Additionally, the relatively small sample size limits the generalizability of our findings. Notably, this study did not directly compare the outcomes with those of other surgical methods. Future case-control studies with larger sample sizes should be conducted to verify the efficacy of this treatment.

## Conclusions

Postoperative outcomes of the locked CW procedure for distal clavicular fractures were favorable. In particular, the ROM improved early after surgery, with nearly full forward elevation achieved six weeks postoperatively. Regarding the concern of pin backout in conventional CW procedures, it remained at <1 mm during the six-month postoperative period, indicating that this complication was successfully overcome. Locked CW is a useful and reliable treatment option for distal clavicular fractures.
